# Morphology of the first-instar nymph and adult female of *Kermes echinatus* Balachowsky, with a comparison to *K. vermilio* Planchon (Hemiptera, Coccoidea, Kermesidae)

**DOI:** 10.3897/zookeys.246.3766

**Published:** 2012-11-29

**Authors:** Malkie Spodek, Yair Ben-Dov

**Affiliations:** 1 Department of Entomology, Volcani Center, Agricultural Research Organization, POB 6, Bet Dagan 50250, Israel; 2 Department of Entomology, Robert H. Smith Faculty of Agriculture, Food and Environment, The Hebrew University of Jerusalem, POB 12, Rehovot 76100, Israel

**Keywords:** Scale insect, *Quercus*, evergreen oaks, Kermesidae, morphology, red dye

## Abstract

Thefirst-instar nymph and the adult female of *Kermes echinatus* Balachowsky (Hemiptera, Coccoidea, Kermesidae) are described and illustrated. This species is compared with *Kermes vermilio* Planchon, a morphologically similar species known in the Palaeractic region.

## Introduction

The scale insect family Kermesidae (Hemiptera, Coccoidea) develops and feeds exclusively on Fagaceae trees (Ben-Dov et al. 2012). This scale insect family is composed of one hundred species distributed among ten genera and they are currently known from the Nearctic, Oriental and Palaearctic regions of the world. *Kermes* Boitard is the principal genus in the European and Mediterranean regions. In these regions, twenty species of *Kermes* have been recorded, all off deciduous and evergreen oak trees (*Quercus*) (Ben-Dov et al. 2012). Individual insects develop mainly in bark crevices and on small twigs and branches (Sternlicht 1969, Bullington and Kosztarab 1985, Hu 1986, Podsiadlo 2005a). Most Kermesidae are not known to cause any visible injury to their host trees. There are however some reports of heavy infestations that can lead to branch dieback, flagging, reduced growth rates and occasionally tree death (Kozár 1974, Hamon 1977, Solomon et al. 1980, Viggiani 1991, Pellizzari et al. 2012).

Seven Kermesidae species, belonging to two genera, *Kermes* Boitard and *Nidularia* Targioni Tozzetti, have been described or recorded from Israel off *Quercus* sp. (Ben-Dov et al. 2012). Two species: *Kermes greeni* and *Kermes nahalali*,were described from the post-reproductive adult female (Bodenheimer 1931); three species: *Kermes echinatus*, *Kermes palestiniensis* and *Kermes spatulatus*, were described from first-instar nymphs (Balachowsky 1953); one species, *Kermes bytinskii*,was described from the adult female and all nymphal instars by Sternlicht (1969). The adult female of *Nidularia balachowskii* was described from Turkey (Bodenheimer 1941) and later was recovered in Israel (Bodenheimer 1944).

*Kermes echinatus* Balachowsky is one of six *Kermes* species found in Israel (Ben-Dov et al. 2012). To date, this species has only been reported off the evergreen oak, *Quercus coccifera* L., from one location in the Lower Galilee in Israel.

Some scale insects have been known as sources of red dye used in the textile, art and wine industries in the Mediterranean, Middle East and Central Asia regions (Leonardi 1920, Donkin 1977, Sarkisov 1984, Cardon 2007) for many centuries. Scholars have suggested that the red dye used for both secular and ritual purposes in Israel during ancient times apparently had been imported from neighboring countries where such dye producing scale insects as *Porphyrophora hameli* Brandt and *Kermes vermilio* Planchon are known (Sandberg 1997). However, Amar et al. (2005) extracted red dye from both the adult females and eggs of *Kermes echinatus* and chemically analyzed it and suggested that *Kermes echinatus* might be the “Tolaat Shani” (scarlet worm in Hebrew), an animal mentioned in the bible used for dye extraction during the period of the second Holy Temple Period (70 A.D.) in Israel.

Balachowsky (1953) described and illustrated the morphology of the first-instar nymph of *Kermes echinatus* from specimens that were collected off *Kermes coccifera* from Nahalal forest located in the Lower Galilee in Israel. He also provided a brief description of the adult female, which included its color, shape and body dimensions.He compared the morphology of *Kermes echinatus* with the first-instar nymph of *Kermes vermilio*, another Palaearctic *Kermes* species not present in Israel, and concluded that “both species share general topography and structure of characters… therefore *Kermes echinatus* is the eastern neighbor of *Kermes vermilio*”.

The first-instar of both *Kermes echinatus* and *Kermes vermilio* are easily distinguishable from other Mediterranean and European *Kermes* species due to the presence of conical, spine-like marginal setae (Balachowsky 1953, Pellizzari et al. 2012). The first-instars of other Palaearctic *Kermes* species possess hair-like, spatulate or club-shaped marginal setae (Kuwana 1931, Balachowsky 1950, Sternlicht 1969, Hu 1986, Liu and Shi 1995, Podsiadlo 2005b). To date there has been no detailed taxonomic description of the adult female of *Kermes echinatus*. This study presents a description of the adult female and a redescription of the first-instar nymph of *Kermes echinatus*. We also compare the general appearance and morphology of both stages with *Kermes vermilio*.

## Materials and methods

### Specimen collections

Between 2010 and 2012, we collected specimens of *Kermes echinatus* off the evergreen oak, *Quercus calliprinos* Webb, from forests in the Golan Heights, the Western, Upper and Lower Galilee regions and the Judean Mountains in Israel. The collection site at Timrat in the Lower Galilee is three km from Nahalal, the type locality of *Kermes echinatus*, and therefore we consider these specimens to be topotypic material. Some of the first-instar nymphs examined in this study emerged from females that were kept in sealed glass containers in the laboratory and other specimens were recovered from thin branches or from trunks of trees.

### Identification and morphological observations

Specimens were processed and mounted on microscope slides according to the methods outlined by Ben-Dov and Hodgson (1997). Illustrations of the adult female and the first-instar nymph of *Kermes echinatus* are generalizations of several specimens, showing the dorsum on the left and the venter on the right, with enlargements of important structures arranged around the central drawing. The structures are not drawn to the same scale between each other. Terms for morphological features follow, chiefly those of Bullington and Kosztarab (1985), Baer and Kosztarab (1985) and Hodgson (1994). Measurements of specimens and of morphological structures were made using an ocular micrometer on an Olympus BX51 phase contrast microscope. Measurements of structures are given in millimeters (mm) or microns (µm). Body length was measured from the farthest points of the head to the posterior end of the body and body width was the greatest width. Setal lengths were measured from the base of the seta to the apex, i.e. excluding the setal socket. Fresh topotypic specimens collected by us in Israel plus one syntypic first-instar nymph were used for the descriptions. The frequency of each structure is given for the entire body. The range is taken from twenty specimens.

Abbreviations of specimen depositories are as follows: **BMNH -** The British Museum (Natural History), London, U.K.; **ICVI -** Coccoidea Collection, Department of Entomology, Agricultural Research Organization, Bet Dagan, Israel; **MNHN -** Museum National d’ Histoire Naturelle, Paris, France.

## Results

### Kermes echinatus

Balachowsky

http://species-id.net/wiki/Kermes_echinatus

Kermes echinatus Balachowsky, 1953:181

#### Note.

This species was originally described from the first-instar nymph collected from Israel, Nahalal forest, off *Quercus coccifera*.

#### Material examined.

**Adult female of *Kermes echinatus*.** Israel: All material was collected off *Quercus calliprinos* by M. Spodek. At least twenty specimens were examined and all material is deposited in ICVI. Alonei Abba Reserve, 19.vi.2011, 26.vi.2011, 3.vi.2012 (MC:530, C:4999), MC:711); Eilon, 19.vi.2011, 22.vi.2011, 26.vi.2011, 3.vi.2012 (MC:533, MC:542, C:4998), MC:692); Nahal Dolev Reserve, 27.vi.2010, 17.vi.2011, 8.vi.2012, 15.vi.2012, 22.vi.2012 (MC:261, MC:699, MC:528, MC:695, MC:709); Hanita, 6.vi.2010 (MC:227); Mas’ada, 4.vii.2010 (MC:285); Nebi Hazuri, 4.vii.2010, 6.vii.2011 (MC:288, MC:556).

#### First-instar nymph of *Kermes echinatus*.

Israel: Syntype (ICVI C:3691, MNHN 1065-8), Nahalal Forest, *Quercus coccifera* 10.v.1950, Bytinski-Salz; All non-type material was collected off *Quercus calliprinos* by M. Spodek, unless otherwise stated; at least twenty specimens were examined and all material is deposited in ICVI. Alonei Abba Reserve, 15.vii.2010, 26.vi.2011, 15.vii.2012 (MC:289, MC:559, MC:719); Eilon, 26.ix.2010, 21.iv.2011, 17.vi.2011, 1.vii.2011, 22.vii.2012 (MC:306, MC:486, MC:499, MC:550, MC:718); Hanita, 13.iii.2011 (MC:457); Nahal Dolev, 22.viii.2010, 8.viii.2011, 1.vii.2012 (MC:293, MC:562, MC:717); Nebi Hazuri, 17.viii.2000, Y. Ben-Dov (C:3409), 4.vii.2010, 6.vii.2011, 17.vii.2011, (C:4818, C:5003, MC:561); Neve Zuf, 10.vii.2000, 18.vii.2003, Y. Ben-Dov (C:4752, C:4751); Timrat, 21.vii.2011, 25.iii.2012 (MC:563, MC:651).

#### Comparative material examined.

**Adult female of *Kermes vermilio*.**France: Corsica, *Quercus ilex*, 7.vi.1999, J. Casevitz-Weal (2 specimens, ICVI C:3277); Le Vert Lasalle, *Quercus coccifera*, 7.v.2007, D. Cardon (3 specimens, ICVI C:4257); Italy: Portofino, *Quercus ilex*, 27.v.1971, D. Matile-Ferrero (2 specimens, MNHN 4594-2), Pistoia, *Quercus ilex*, 13.viii.1986, A. Belcari (2 specimens, MNHN 10732-1), Bitonto (Bari), *Quercus ilex*, 25.vii.2012, F. Porcelli (10 specimens, ICVI C-5132); Spain: Mieras (Gerona), *Quercus coccifera*, 7.v.1987, A. Verhecken (1 specimen, MNHN 11526-1).

#### First-instar nymph of *Kermes vermilio*.

France: Le Vert Lasalle, *Quercus coccifera*, 24.vi.2007, D. Cardon (47 specimens, ICVI C:4272); Italy: Pistoia, *Quercus ilex*, 13.viii.1986, A. Belcari (6 specimens, MNHN 10732-3), Bitonto (Bari), *Quercus ilex*, 28.viii.2012, F. Porcelli (20 specimens, ICVI C-5133)**;** Spain: Mieras (Gerona), *Quercus coccifera*, 7.v.1987, A. Verhecken (1 specimen, MNHN 11526-2)**.**

#### Description adult female.

**Figure 1.**
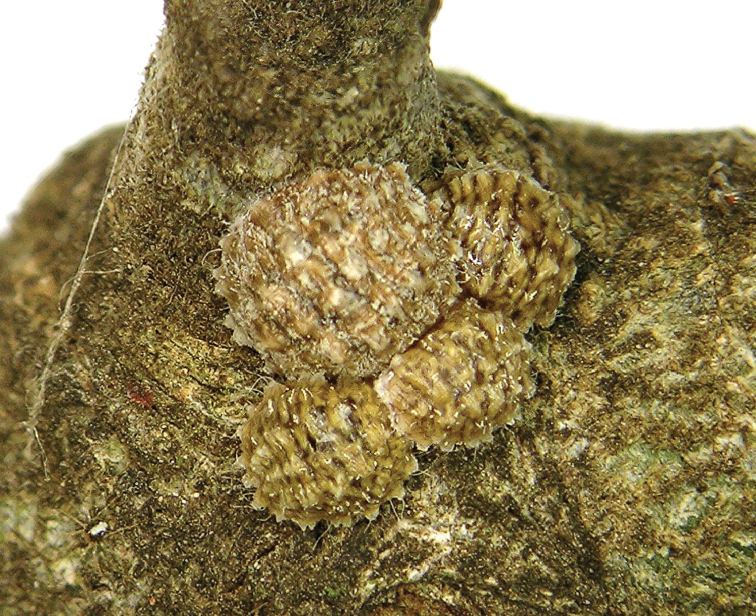
*Kermes echinatus* Balachowsky young adult female, general appearance.

**Figure 2.**
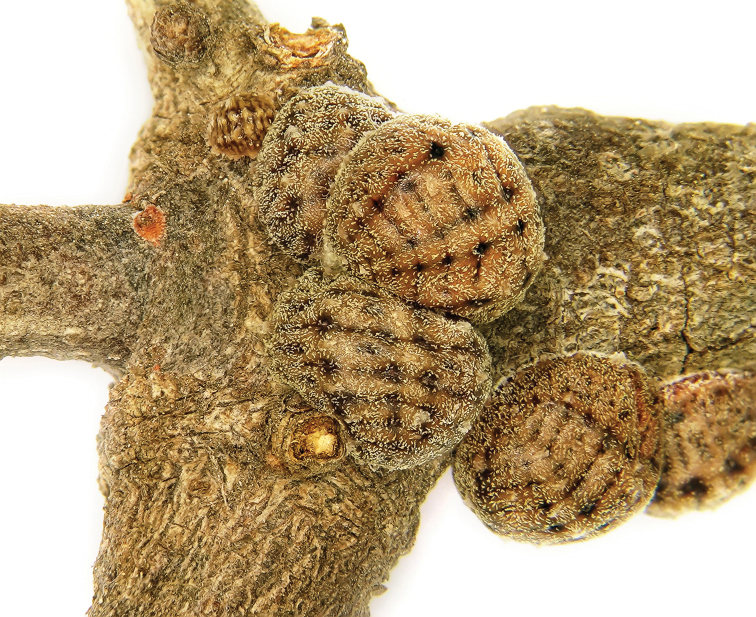
*Kermes echinatus* Balachowsky mature reproductive female, general appearance.

**Figure 3.**
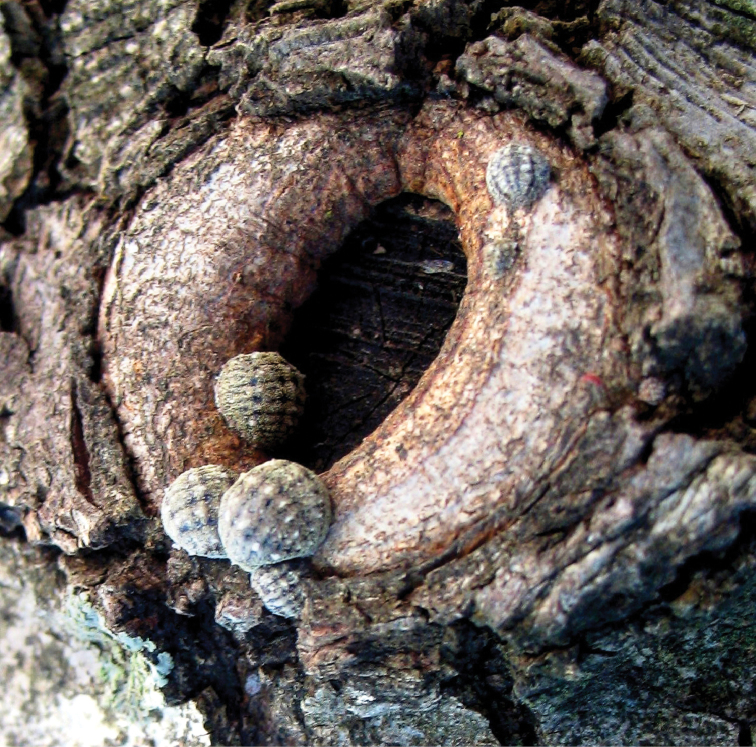
*Kermes echinatus* Balachowsky gravid females on tree trunk, general appearance.

**Figure 4.**
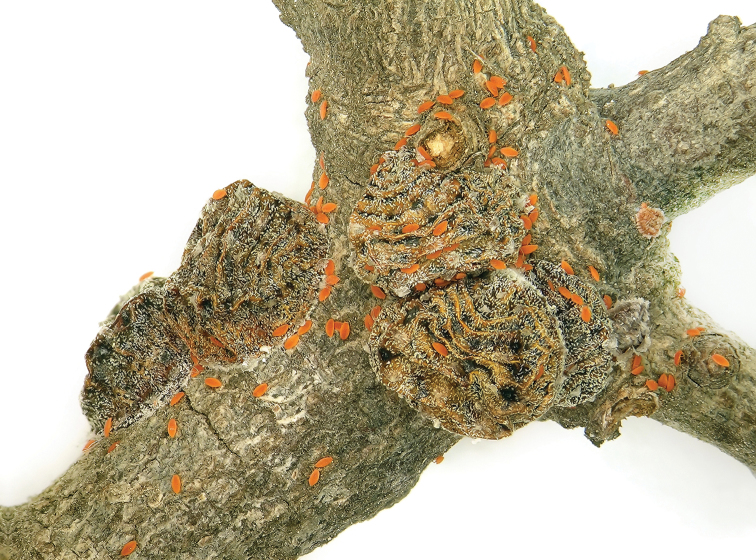
*Kermes echinatus* Balachowsky female with emerging first-instar nymphs.

**General appearance.** Young, pre-reproductive adult: Oval, soft and slightly convex; dorsum brownish-grey with 4 or 5 black longitudinal and 6–9 black transverse lines formed of dots and lines; 2.5–3.2 mm long and 2–3 mm wide (Fig. 1). Fully-mature reproductive female highly convex; dorsum brownish-grey with black, longitudinal and transverse lines; body tapering posteriorly (Figs 2, 3). Post-reproductive female oval and moderately convex, 2.9–4.4 mm long, 2.7–5.1 mm wide and 3.2–4.8 mm high; dorsum sclerotized; red with 6–9 black, transverse black lines represented as reticulated folds (Fig. 4).

**Figure 5.**
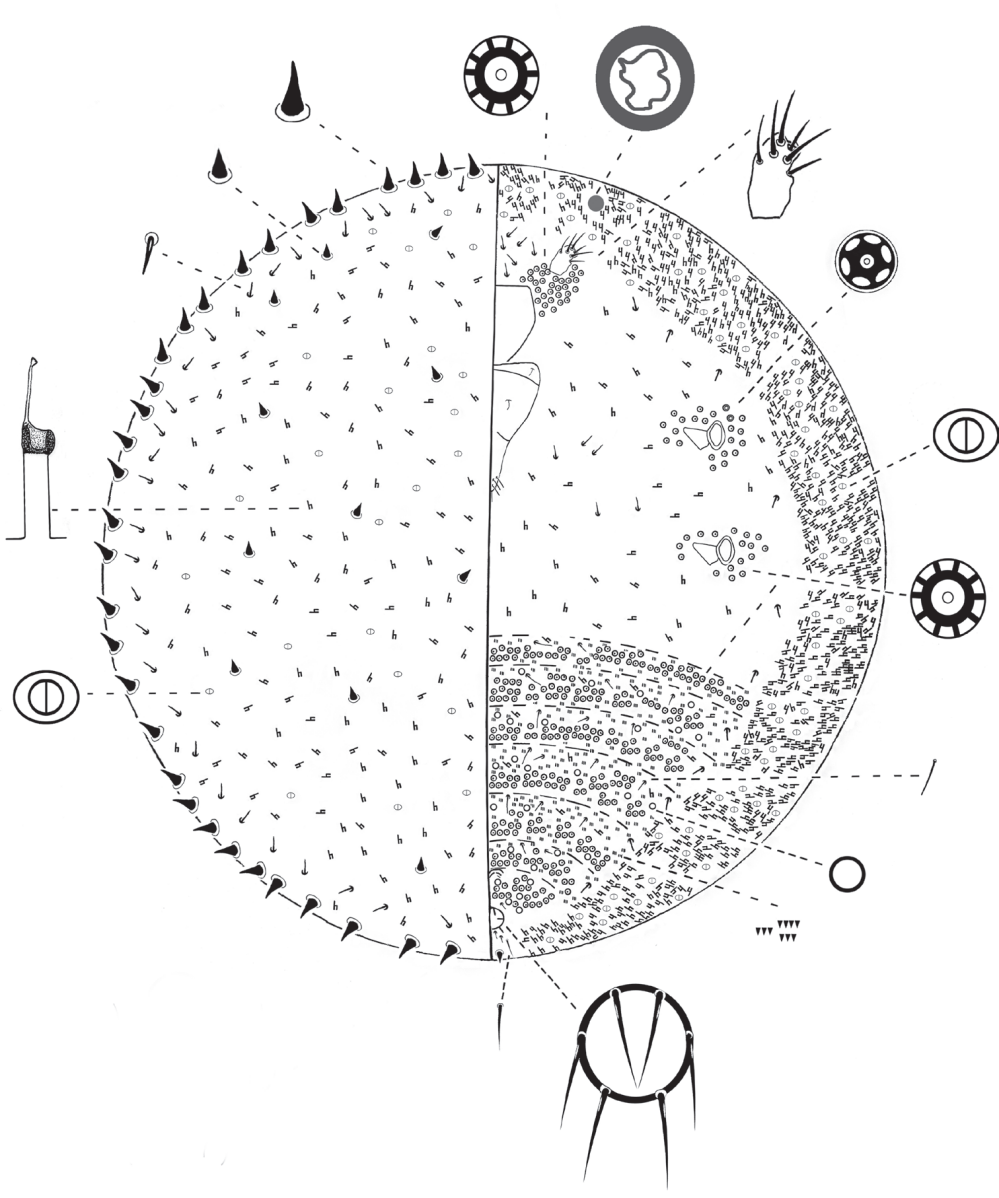
*Kermes echinatus* Balachowsky adult female.

**Slide-mounted** **adult female**. 2–3 mm long and 2–2.8 mm wide (Fig. 5).

**Margin. Marginal setae** conical, 12–13 µm long and 10–11 µm wide at base; arranged in a single row of 30–38 setae on each margin.

**Dorsum. Dorsal setae** hair-like, 7–9 µm long, in submarginal band from cephalic tip of body to posterior end of body, about 28–33 on each side. **Conical setae,** similar in shape to marginal setae, randomly placed on dorsum, with 7–11 setae on each side; each seta 10–13 µm long and 7–10 wide at base. **Bilocular pores** oval with a sclerotized rim, each 3 µm long and 2 µm wide; present throughout. **Tubular ducts** diffused throughout dorsum; each with outer ductule 12–17 µm long, inner ductule 10–15 µm long and with a sclerotized cup 5 µm diameter.

**Venter. Eyes** circular, 20–25 µm diameter, each placed anterolaterally to each antenna. **Legs** absent. **Antenna** each 1-segmented; 26–35 µm long, 20–31 µm wide**;** eachbearing 5–8 fleshy setae; each antennae is surrounded by a group of 40–45 multilocular pores; each pore 7–8 µm diameter with 10 loculi. **Clypeolabral shield** 235–250 µm long, 212–225 µm wide. **Labium** 3-segmented, triangular, 160–175µm long, 110–135 µm wide; labial setae as follow: basal segment with 2 setae, 5–8 µm long; medial segment with 2 setae, 12–20 µm long; apical segment with 4 setae; 6 apical setae, 10–12 µm long and 2 subapical seta, 7–8 µm long. **Mesothoracic and metathoracic spiracles** subequal in size; peritreme 50–68 µm long and 30–37 µm wide; pores with 10 loculi and 8 µm wide in a group of 15–22 locular pores laterad to each spiracle; also with 2 pores with 6 loculi, each 6µm diameter, laterad to each anterior peritreme. **Tubular ducts** present in a complete, dense submarginal band about 11 ducts wide and also sparsely throughout rest of venter; each duct with outer ductule 10–16 µm long; inner end of outer ductule with a sclerotized cup, 4–5 µm diameter, and inner ductule 11–15 µm long. **Multilocular pores** each diameter 10 µm with 10–12 loculi, arranged in 2–3 transverse rows on each abdominal segment; with a total of 114–120 pores on each segment; also with a group of 52–56 pores just posterior to vulva. **Bilocular pores** each3 µm long and 2 µm wide, interspersed between tubular ducts in submarginal band. **Simple pores** 2 µm diameter with a sclerotized rim, interspersed between multilocular pores on abdomen. **Ventral setae** 7–12 µm long, distributed as follows: about 12 setae just anterior to clypeus between antennae; about 8 setae on median and submedian areas of thorax; about 11 setae mesad to each submarginal band of tubular ducts, in a line from antennae to anal ring; 6 or 8setae, present in a band along each abdominal segment; plus 2 setae 20–25 µm long, placed medially on each abdominal segment. **Microspines** each 1–2 µm long, in groups of 3–5, in 3–8 rows on each abdominal segment. **Anal ring** ventral, forming a complete sclerotized circle; diameter 42–60 µm; cells absent; with 6 setae, each 25–40 µm long. **Other ventral setae** 1 pair of setae, each 10–12 µm long, present just anterior to anal ring; 2 pairs of setae, each 10–12 µm long, present posteriorly to anal ring; 1 pair of stout conical setae (similar in shape to marginal spinose setae but shorter), each 10–12 µm long and wide, present on venter slightly above posterior margin; and 1 pair of apical setae, each 33–35 µm long.

#### First-instar nymph.

**Figure 6.**
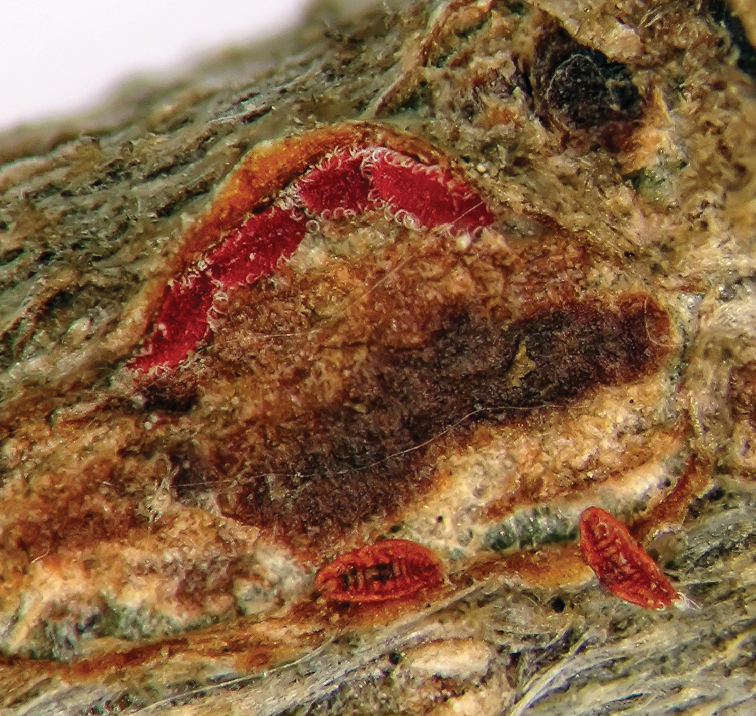
*Kermes echinatus* Balachowsky first-instar nymph, general appearance.

**General appearance.** Dorsum and venter red, body oval and tapering posteriorly, 0.37–0.44 mm long and 0.14–0.2 mm wide. Each with a fringe of curly white wax on margins once first-instars settle on branch for feeding (Fig. 6).

**Figure 7.**
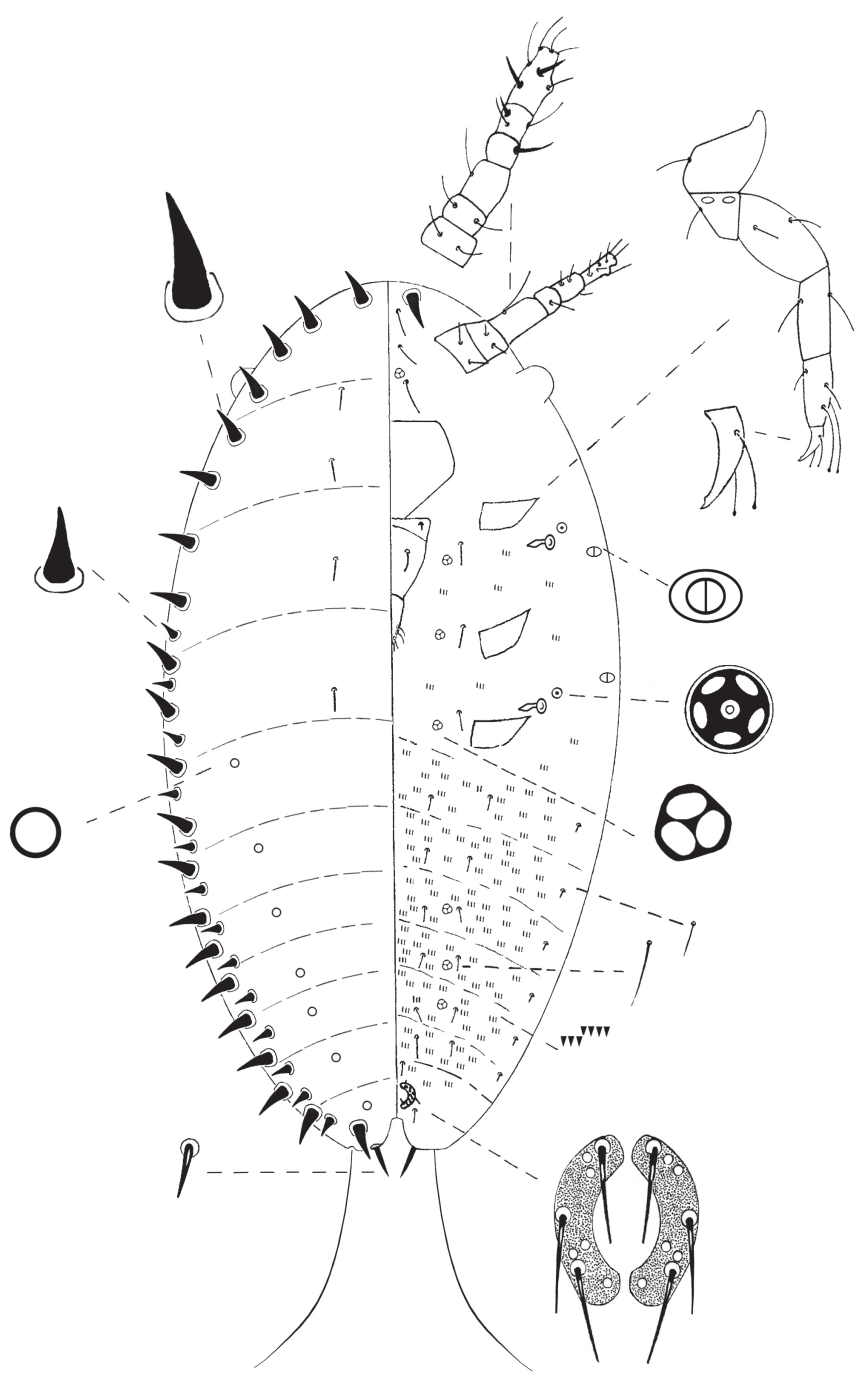
*Kermes echinatus* Balachowsky first-instar nymph.

**Mounted** **specimen**. Oval, 0.45–0.49 mm long and 0.20–0.25 mm wide (Fig. 7).

**Margin**. **Marginal setae** conical and slightly curved apically, each 10–13 µm long and 5 µm wide at base, in a complete line of 17–22 on each side, smaller conical setae, not-curved, each 5–8 µm long and 3–5 µm wide at base, in a submarginal row of 12–14 setae extending from mesothorax to anal lobe.

**Dorsum.** Derm membranous; intersegmental lines observable. **Dorsal setae** 8, each 6–8 µm long, placed in 2 submedian, longitudinal rows on thorax. **Simple pores** circular, 14, eachabout1 µm diameter, placed in 2 submarginal, longitudinal rows on abdomen.

**Venter. Antennae** each 6-segmented; total length 102–110 µm; with segment III and VI longest; setal distribution as follows: scape and pedicel each with 2 thin, hair-like setae; segment III with 1 long thin, hair-like seta; IV with 1 fleshy setae; V with 1 fleshy seta and 2 hair-like setae; apical segment with 2 fleshy setae and 5 hair-like setae. **Legs** well-developed; measurements of hind legs (length in µm); coxae 25–30, trochanter + femur 68–70, tibia 33–38, tarsus 45–50, claw 15–20; total leg length 187–200 µm; trochanter with 2 oval, sensory pores on each side, each pore 3 µm long and about 2 µm wide; setae present on all leg segments; tarsal digitules each 25–30 µm long and knobbed apically, extending beyond apex of claw;claw digitules knobbed apically, each 15–20 µm long; each claw with a single denticle near tip. **Clypeolabral shield** well-developed; 68–75 µm long and 63–75 µm wide. **Labium** 3-segmented, triangular, 75–83 µm long and 45–47 µm wide; labial setae as follows: basal segment with 1 setae, rarely 2 setae, 5–8 µm long, median segment with 2 hair-like setae on dorsal surface, 12–13 µm long, apical segment with 6 subapical setae, each 16–20 µm long and 2 apical setae 10–12 µm long. **Spiracles** subequal in size; each peritreme 3–5 µm diameter; apodeme crescent shaped, 13–15 µm long; each spiracle with 1 quinquelocular pore, 5 µm diameter, placed anterolaterally. **Trilocular pores**, each about 3 µm wide, distributed as follows: 2 pores between scape just anterior to clypeus; 1 mesad to each coxa, and 2 submedially on abdominal segments V–VII. **Bilocular pores** oval, 4 total, each 3 µm long and 2 µm wide, present between margin and each spiracle. **Ventral** **setae** interantennal setae 6, each 38–45 µm long, in an longitudinal line medially between scapes; also 2 conical setae, about 14–16 µm long and 5 µm wide at base, on anterior apex of head; 1 seta 10–11 µm long, mesad to each coxa associated with each trilocular pore; and 6 longitudinal lines of setae on abdomen; with 2 medial, 2 submedial and 2 submarginal seta per segment; medial and submedial setae each 10–15 µm long, and submarginal setae 5–6 µm long. **Microspines** each about 3 µm long, arranged in groups of 3 or 4 in 2 transverse rows on each abdominal segment and sparsely on thorax. **Anal ring** located ventrally; composed of 2 semi-circles; diameter 20–25 µm; each half circle with 4–6 cells and 3 pointed setae, each 13–18 µm long. **Other setae** with a pair of setae, each 15–18 µm long, anterior to anal ring, and a pair, each 15–20 µm long, latero-posteriorly to anal ring. **Anal lobes** slightly developed; inner margin of each lobe with 1 pointed seta, 10–13 µm long and 2–3 µm wide, and 1 very long, flagellate seta apically 220–275 µm long.

## Discussion

Prior to this study, *Kermes echinatus* had only been reported off the evergreen oak, *Quercus coccifera*, from Nahalal forest, located in the Lower Galilee of Israel (Balachowsky 1953). During the 2010 to 2012 surveys of Kermesidae throughout the country, specimens were recovered off *Quercus calliprinos* trees in the Golan Heights, Western, Upper and Lower Galilee regions, as well as the Judean Mountains. It is widely accepted by botanists that *Quercus calliprinos* is probably an East Mediterranean subspecies of, or a vicariad species to, *Quercus coccifera*, which is distributed in the Mediterranean territories of Europe (Zohary 1973, Jalas and Suominen 1976).

The present description of the first-instar nymph of *Kermes echinatus* agrees well with that of Balachowsky (1953). However our redescription includes several features that were not indicated in the original description but were observed by in us in fresh and type material. These features are the presence of: (i) dorsal submedial setae; (ii) ventral bilocular pores; (iii) a claw denticle on each leg; (iv) and microspines dispersed on thoracic and abdominal regions.

Balachowsky (1953) observed two main differences between the morphology of the first-instar nymph of *Kermes echinatus* and *Kermes vermilio*: (i) dorsal simple pores present on *Kermes echinatus* but absent on *Kermes vermilio* and (ii) the structure and arrangement of the marginal conical setae. In *Kermes echinatus*, the conical marginal setae are slightly longer and curved compared to those of *Kermes vermilio*. Balachowsky considered that the marginal setae of *Kermes vermilio* were of one length and arranged in two rows while those of *Kermes echinatus* were arranged in one row, but we found that first-instar *Kermes echinatus* also had two rows of conical marginal setae but that they differed in size and shape.

In addition, we observed other distinguishing features between the two species and these are summarized in Table 1. Pellizzari et al. (2012) added that the living specimens of *Kermes vermilio* are orange-red with yellow legs, whereas we noted that the first-instar nymphs of *Kermes echinatus* are red. We also noted a small denticle on each claw of *Kermes echinatus*. These were considered to be absent on *Kermes vermilio* by Balachowsky (1950) and Pellizzari et al. (2012).

**Table 1.** Comparison of some characters of the first-instar nymph of *Kermes echinatus* and *Kermes vermilio*.

Character	*Kermes echinatus*	*Kermes vermilio*
Dorsal simple pores	present	absent
Dorsal bilocular pores	absent	present
Locular pores associated with prothoracic spiracles	1 pore, 5 loculi	1 pore, 5 loculi and 7 loculi
Arrangement of marginal setae	2 rows	2 rows
Type of conical marginal setae	2 types	1 type
Denticle on claw of legs	present	absent

**Table 2.** Comparison of some characters of the adult females of *Kermes echinatus* and *Kermes vermilio*.

Character	*Kermes echinatus*	*Kermes vermilio*
Marginal and dorsal conical setae	present	present
Hair-like setae in submarginal band on dorsum	present	absent
Conical setae in submarginal band on venter	absent	present
Legs	absent	absent
Position of anal ring	ventral	ventral
6 -locular pores associated with prothoracic spiracles	2 pores present	absent
Setae on anal ring	present	absent
Cells on anal ring	absent	present
Simple pores on abdomen	present	absent
Multilocular pores posterior to vulva	present	absent

The general appearance of young females and fully-grown reproductive females of *Kermes echinatus* differs from that of *Kermes vermilio*. The young female of *Kermes echinatus* is slightly convex, has a brownish-grey dorsum with 4 or 5 black longitudinal and 6–9 black transverse lines composed of dots and lines. The young female of *Kermes vermilio* is reddish without transverse and longitudinal lines. The fully-grown reproductive female of *Kermes vermilio* has been described as dark red or brown covered with a fine, white or pale grey mealy wax (Pellizzari et al. 2012). In contrast, the fully-grown female of *Kermes echinatus* is not covered in wax and has transverse and longitudinal black lines on its dorsum. Both species at this stage are highly convex and subspherical.

The morphological features of the adult female of *Kermes echinatus* and *Kermes vermilio* are similar and are summarized in Table 2. Some of the shared features are the following; (i) dorsal and marginal conical setae; (ii) absence of legs; (iii) presence of numerous multilocular pores on abdominal segments as well as surrounding the antennae and spiracles; (iv) one-segmented antennae with fleshy setae; and (v) the anal ring located ventrally in both species. The most distinguishing feature of *Kermes echinatus* is the anal ring which has six setae and no cells whereas the anal ring of *Kermes vermilio* has cells but no setae. Some other differences between the two species are that *Kermes echinatus* has less conical setaeon its margins and dorsum compared to *Kermes vermilio*. *Kermes echinatus* has 30–38 setae on each half margin compared to 73–133 in *Kermes vermilio*. *Kermes echinatus* has 7–11 dorsal setae compared to about 70 dorsal setae in *Kermes vermilio*. Ventral loculate pores are only found on the abdominal segments and surrounding the spiracles in *Kermes echinatus* in contrast to *Kermes vermilio*, where they extend onto the metathorax from the abdomen.

## Conclusion

This paper describes the adult female of *Kermes echinatus* for the first time and redescribes the first-instar nymph. The general appearance and morphological features of *Kermes echinatus* and *Kermes vermilio*, two species that have been linked to sources of red dye in the Palaerarctic region, are compared. Distinguishing characters of the first-instar nymph and female of *Kermes echinatus* are presented. *Kermes echinatus* has only been recorded in Israel to-date and is one of seven species of Kermesidae occurring there.

## Supplementary Material

XML Treatment for Kermes echinatus
